# The Increasing Trend in Global Ranking of Websites of Iranian Medical Universities during January 2012–2015

**Published:** 2017-08

**Authors:** Nahid RAMEZAN GHORBANI, Yousef FAKOUR, Seyed Ali NOJOUMI

**Affiliations:** 1. Dept. of Development and Cooperation of Information and Scientific Publications, Undersecretary for Research and Technology, Ministry of Health and Medical Education, Tehran, Iran; 2. Deputy for Research and Technology, Ministry of Health and Medical Education, Tehran, Iran; 3. Microbiology Research Center, Pasteur Institute of Iran, Tehran, Iran

**Keywords:** Webometrics, Ranking, Universities of medical sciences, Iran

## Abstract

**Background:**

Researchers and academic institutions need assessment and rating to measure their performance. The criteria are designed to evaluate quality and adequacy of research and welcome by most universities as an international process to increase monitoring academic achievements. The study aimed to evaluate the increasing trend in global ranking of Iranian medical universities websites emphasizing on comparative approach.

**Methods:**

This is a cross-sectional study involving websites of Iranian medical universities. Sampling was conducted by census selecting universities affiliated to the Ministry of Health in webometrics rating system. Web sites of Iranian medical universities were investigated based on the webometrics indicators, global ranking as well as the process of changing their rating. Universities of medical sciences were associated with improved ratings in seven periods from Jan 2012 until Jan 2015.

**Results:**

The highest rank was in Jan 2014. Tehran University of Medical Sciences ranked the first in all periods. The highest ratings were about impact factor in universities of medical sciences reflecting the low level of this index in university websites. The least ranking was observed in type 1 universities.

**Conclusion:**

Despite the criticisms and weaknesses of these webometrics criteria, they are critical to this equation and should be checked for authenticity and suitability of goals. Therefore, localizing these criteria by the advantages model, ranking systems features, continuous development and medical universities evaluation based on these indicators provide new opportunities for the development of the country especially through online media.

## Introduction

Researchers and academic institutions increasingly need assessment and rating as a measure of individual and organizational performance. This is an international process. Increasing surveillance on the academic achievements through the assessment of excellence in research (in Australia), UK research assessment, Performance-Based Research Funding in New Zealand have attracted the attention of the universities. All these criteria manifest the quality and efficiency of research (1). Rating systems have been the focus of discussions among the higher education leaders since 1980 (2). A study was conducted about the ranking of medical schools to express restrictions on the ranking for specific programs (3). The cases discussed consist of strengths, mission of the university, their impact on the society in which they serve and their weaknesses. This study stressed the importance of the mission of universities and challenges for the leaders and teaching hospitals based on reliable data and the quality of health promotion programs for everyone. Development of university rankings is a joint action, especially in the English-speaking countries. Rankings are mainly used for items such as comparative analysis of internal or international institutions, improved policy analysis and/or economic investment by state, provision of subsidies to public and private investment purpose and to help in the process of university selection for perspective students.

The development of this ranking can lead to the development of scientific institutions while providing support for investment in research (1).

Today, the web is an important tool for formal and informal communication and cooperation among individuals including researchers. Increased number of indexed web pages paved the way for the emergence of the methods and measures of web resources (4).

Website and access to the web visibility of the university is one of the important factors for the success of the university; therefore, evaluation and ranking of universities’ websites is as crucial as evaluation and ranking of research and educational activities of universities. Thus, evaluation of universities on the web is one of the priorities of the country as a tool for evaluating the academic and research performance of universities (5).

Ranking systems of university websites recognize internet as a source for large volumes of documents and a tool to disseminate and access knowledge. Rankings of Shanghai, Times and QS are some of the most important systems of the world university rankings. Indicators such as scientific achievements, professional judgment score, quality of education, the number of foreign students, winners of the Nobel Prize are evaluated in most of these systems. Webometrics Rating system examines the overview and comprehensive images of a university (6). It also evaluates academic and educational presence of the universities on the Web through websites. The websites of universities and research centers are classified based on four criteria;

- The number of web pages in the university website
- Total number of PowerPoint, Word and PDF files available on the website of the university
- The qualitative papers of the university among the 10% most cited articles in SCImago website (7).

The number of links to the university website (2015) Webometrics is introduced as the quantitative aspects of the structure and use of information resources, structures and web technologies outlining approaches to information and bibliometrics. It also takes into grant four main areas of analysis of the content and structure of the web, web use and technologies applied to the web. Webometrics may become one of the most interesting research areas for a wide array of electronic information (8, 9).

Thus, considering the importance of academic websites, this study examined websites based on the Webometrics in order to improve in webometrics ranking and to reveal a complete perspective of the status of science and research in the field of health in the country and the presence and success of the universities of medical sciences.

## Methods

In this cross-sectional study, the population of the research included websites of Iranian medical universities. Sampling was conducted by census. In the first stage, all medical universities were recovered in terms of ranking in the webometrics database by selecting the Asia and Iran, respectively. Then, type one, two and three universities of Ministry of Health and Medical Education were separated in webometrics database in terms of rating among the items recovered. Secondly, web content of universities was investigated based on the webometrics indicators (presented in Jan 2015), their national webometrics ranking and changes in their rating at 7 periods (Jan–Jul 2012, Jan–Jul 2013, Jan and Jul 2014 and Jan 2015). The increasing trend in ranking of websites of recovered universities was compared using descriptive statistics (percentage, frequency, Table and Figures). Web ranking of universities in global webometrics ranking system is done on 4 criteria including Presence (the number of web pages in the university website), Openness (total number of Power Point, Word and PDF files available on the website of the university), Excellence (the quality papers of the university among the 10% most cited articles in SCImago website) and Impact (the number of links to the university website).

## Results

Rank status and development of medical universities is as follows with regard to webometrics ranking results in the time of Jan 2012 until Jan 2015:

Medical universities of Tehran, Isfahan, and Shiraz ranked first and third, respectively in Jan 2012. Medical universities of Lorestan (43%), Tehran (35%), Isfahan (31%), Shahrekord (29%), Kermanshah (25%) and Mazandaran (24%) showed considerable growth. The results suggested that 74% of medical universities have been associated with increased global ranking in Jan 2012 and the total number was 35 universities.

Rating of universities in Kerman, Mazandaran, Arak, Kurdistan, Tehran, Kermanshah, Semnan, Birjand, Babol, and Hamadan indicated a growth more than 20% in Jul 2012 compared to the first results of the ranking in Jul 2011. 61.4% of medical universities in 2012 were associated with an increase in global ranking (total: 44 universities).

Three medical universities of Tehran, Isfahan, and Shahid Beheshti received top ratings in Jan 2013 and medical universities of Qazvin, Gonabad, Kurdistan, Hormozgan, Arak and Bushehr showed a growth more than 20% compared to the previous rating. 61.4% of medical universities in 2013 were associated with an increase in global ranking (total: 46 universities).

Three medical universities of Tehran, Shiraz, and Isfahan received top ratings in Jul 2013 and medical universities of Fasa, North Khorasan, and Army indicated a growth more than 20% compared to the previous rating. Twenty-six percent of medical universities in 2013 were associated with an increase in global ranking (total: 46 universities). Medical universities of Dezful and Torbat were also added to the list of webometrics ranking.

Medical universities of Tehran, Shiraz, and Isfahan received top ratings in Jan 2014 and medical universities of North Khorasan, Gonabad, Ahvaz, Army, Lorestan, Zahedan, Qom, Hamadan, Mashhad, and Shiraz showed an increase more than 25% compared to the previous rating. 91.6% of medical universities in Jan 2014 were associated with an increase in global ranking. Medical universities of Isfahan, Arak, and Fasa also showed degradation.

Medical universities of Tehran, Tarbiat Modares, and Shiraz received top ratings in Jul 2014 and medical universities of Ardebil, Modares, Shahrekord, Qazvin, Kashan, Kerman and Shiraz indicated a growth more than 40% compared to the previous rating. 81.2% of medical universities in Jul 2014 were associated with an increase in global ranking. The nine medical universities of Semnam, Zahedan, Sabzevar, Babol, Tavanbakshi, Torbat Heydarieh, Yasouj, Qom, and Dezful also indicated a ranking decrease.

Medical universities of Tehran, Shahid Beheshti, and Isfahan received top ratings in Jan 2015; while, medical universities of Kordestan, Torbat Heydarieh, Rafsanjan and Birjand indicated a growth more than 15% compared to the previous rating. Among 50 universities of medical sciences in the global webometrics ranking, 44% associated with improved global ranking in recent rating. They are generally ranked 37.2% lower than the previous rating. The medical universities of IAU, Shiraz, North Khorasan, Lorestan, Isfahan, Tavanbakshi, Dezful, Zahedan, Hormozgan, Sabzevar, Babol, Kerman, Ilam, Yasouj, Qom, Fasa, Tabriz, Jahrom, Arak, Shahed, Golestan, Zabol, Qazvin, Gonabad, Shahroud, Kermanshah, Ardebil and Hamedan also indicated a backward (Table 1).

**Table 1:** The global rating changes for websites of medical universities according to webometrics rating system (Jan 2012 till Jan 2015)

**Rating of Medical Sciences**	**Name of Medical Science Universities**	**Global rank in Jan 2015**	**Global rank in Jul 2014**	**Global rank in January 2014**	**Global rank in Jul 2013**	**Global rank in Jan 2013**	**Global rank in Jul 2012**	**Global rank in Jan 2012**
1	Tehran	385↑	422↑	615↑	784↓	612↑	656↑	668
2	Tarbiat Modares	603↑	641↑	1392↓	1351↑	2424↑	2512↓	1993
3	Shahid Beheshti	926↑	1053↑	1461↑	1711↓	1350↑	2016↑	2246
4	Isfahan	1094↓	981↑	1259↑	1649↓	1185↑	1893↓	1072
5	Mashhad	1138↑	1151↑	1358↑	1807↓	1690↓	1581↓	1459
6	Shiraz	1259↓	704↑	1184↑	1579↓	1406↓	1390↓	1254
7	Tabriz	1548↓	1458↑	1883↑	2123↓	1958↓	1908↓	1520
8	Kerman	1799↓	1665↑	2859↑	3151↓	2058↑	2242↑	4843
9	Birjand	2287↑	2702↑	2926↑	3860↓	2436↑	4047↑	5761
10	Kordestan	2362↑	3524↑	4212↓	4211↓	3282↑	6042↑	8934
11	Ardebil	2424↓	2406↑	5305↑	6540↓	4193↑	5809↓	3621
12	Zanjan	2483↑	2493↑	3105↑	3686↓	3076↑	4742↓	3910
13	Kermanshah	2566↓	2545↑	3995↑	4293↓	3158↑	3235↑	3518
14	Shahid Sadoughi	2576↑	2602↑	4286↑	5635↓	4613↓	4533↑	5202
15	Hamedan	2577↓	2558↑	4017↑	5436↓	3705↑	4376↑	4575
16	Mazandaran	2667↑	2694↑	3805↑	4350↓	2743↑	4189↑	7005
17	Arak	2756↓	2616↑	4288↓	4286↓	3326↑	5896↑	9349
18	Shahed	2765↓	2670↑	3062↑	3265↑	3672↑	5022	3293
19	Urmia	3075↑	3372↑	4483↑	5563↓	4398↑	6020↑	6534
20	Baqiyatallah	3145↓	3439↑	3645↑	3775↑	4244↑	4884↑	6734
21	Iran	3173	0	0	0	0	0	0
22	Gilan	3191↑	3599↑	4404↑	4889↑	5211↓	5156↑	5315
23	Kashan	3393↓	3429↑	6251↑	7651↓	4476↑	6535↑	8029
24	Golestan	3472↓	3368↑	5081↑	5900↓	3519↑	5047↓	4068
25	Qazvin	3787↓	3698↑	6796↑	8115↓	4732↑	9818	8991
26	Tavanbakhshi	3929↓	3554↑	3253↑	4052↓	3135↑	4213↑	7393
27	Shahrekord	4007↑	4170↑	8470↑	10829↓	6134↑	9762↓	5552
28	Army	4108↑	4160↑	5713↑	8893↑	11358↑	16210↓	14936
29	Qom	4669↓	4377↑	4346↑	8773↓	4698↑	6800↑	7075
30	Jondi Shapour	4677↑	4800↑	6010↑	8285↓	4836↑	5816↓	4458
31	Bushehr	4714↑	4960↑	6118↑	8785↓	6169↑	10703↓	10067
32	Hormozgan	4766↓	4367↑	5429↑	6593↓	4512↑	8095↓	7157
33	Rafsanjan	4821↑	5769↑	6628↑	8552↓	6866↑	7800↓	7929
34	Lorestan	5231↓	4538↑	5635↑	8101↓	4427↑	6506↓	4134
35	Babol	5262↓	4849↑	4436↑	5618↑	6696↑	6702↓	8438
36	Ilam	5404↓	5033↑	6016↑	7491↑	7645↑	8588↑	8596
37	Gonabad	5813↓	5680↑	7350↑	9922↓	6109↑	11490↓	10480
38	North Khorasan	6431↓	5570↑	8308↑	11013↑	17518↓	14096	0
39	Fasa	6673↓	6278↑	8417↓	5716↑	10661	10254↓	9226
40	Zahedan	6869↓	6286↓	5476↑	7887↓	7521↑	6453↑	12969
41	Yasouj	7516↓	7032↓	6802↑	8202↑	9466↑	10807↓	9788
42	Shahroud	7708↓	7591↑	9601↑	12323↓	8372↓	6789↑	7954
43	Jahrom	8925↓	8430↑	10114↑	10645↑	11358↓	8510↓	7753
44	Sabzevar	9875↓	9086↓	8023↑	8869↓	5670↑	8429↑	9050
45	Semnan	10444↑	11554↓	6208↑	6244↓	5868↑	5884↑	8285
46	Zabol	10469↓	10169↑	10269↑	12767↓	9925↓	9458↑	10506
47	Alborz	10514↓	0	0	0	0	0	0
48	IAU	11976↓	6025↑	9353↑	9735↑	11284↑	11555↓	9803
49	Torbat Heydarieh	12725↑	15297↓	14159↑	18437	0	0	0
50	Dezful	13849↓	12579↓	12492↑	15592	0	0	0

* *0= Data Unavailable*

**Fig. 1:**
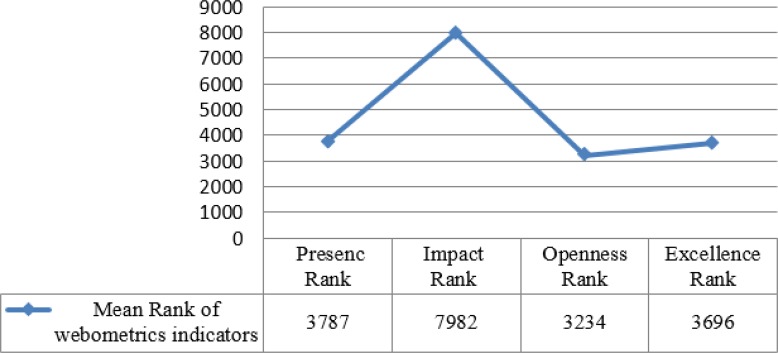
Ranks mean of webometrics indicators of medical sciences universities in Iran

The highest index in rating is observed in universities of Tehran, Birjand and Shiraz, respectively and the lowest rank is seen in universities of Torbat Heydarieh and IAU. The highest impact indicator is observed in universities of Tehran, Tarbiat Modares, and Shahid Beheshti; whereas, the lowest impact indicator is seen in Sabzevar University. The highest rank in the field of excellence indicator is observed in the universities of Tehran, Tarbiat Modares, and Shahid Beheshti. The average index of rating showed that the highest rank results from impact factor. It is determined by numeration of external links to university subdomains. This obviously is more important than the other indices with a 50% of allocation. External link indicates the reputation of the organization, academic performance, value and usefulness of services presented in web pages. They are obtained by two important suppliers named Majestic SEO and Ahrefs given that the highest rating reflects a low level of this index in the university websites (Fig. 1).

**Fig. 2:**
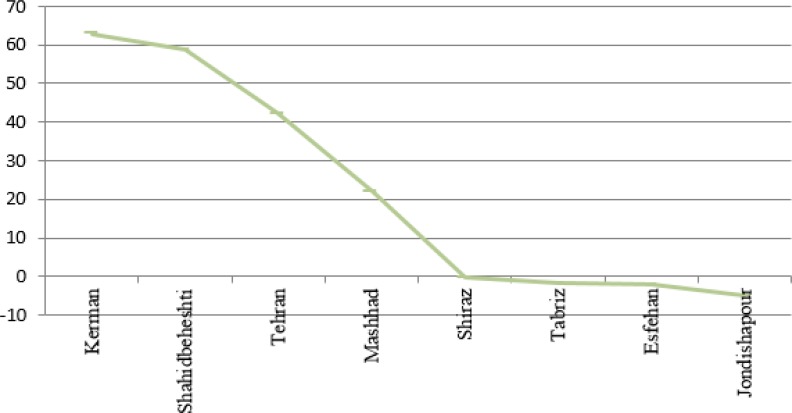
Comparison growth percentage of webometrics rank of medical sciences university’s Type 1 in Iran during Jan 2012–2015

The study of growth rate in type 1 medical universities indicated that the highest growth rate is seen in medical universities of Kerman, Shahid Beheshti, and Tehran, while, medical universities of Ahvaz and Isfahan faced with negative growth. If severance of years is considered, the greatest growth in Jan 2015 is seen in Shahid Beheshti University of Medical Sciences (12%) and the highest negative growth is seen in Shiraz University of Medical Sciences (79%). The greatest growth in Jul 2014 is seen by Kerman University of Medical Sciences (42%) and the highest negative growth is seen in Mashhad University of Medical Sciences (15%). The highest growth in Jan 2014 is manifested in Ahvaz University of Medical Sciences (27%), whereas, the highest negative growth is seen in Kerman University of Medical Sciences (9%). All universities had negative growth in Jul 2013 and the highest negative growth is seen in Ahvaz. %). The best growth in Jan 2013 is seen in Isfahan University of Medical Sciences (37%); whereas, medical universities of Shiraz, Tabriz, and Mashhad presented a negative growth. The greatest growth in Jul 2012 is seen in Kerman University of Medical Sciences (54%); unlike this, the highest negative growth is seen in Isfahan University of Medical Sciences (77%). If we consider the whole period of rating, the greatest growth in Jul 2012 is manifested in Kerman University of Medical Sciences and the highest negative growth is seen in Shiraz University of Medical Sciences in Jan 2015 (Fig. 2).

The study of growth rate in type 2 medical universities indicated that the highest growth rate is seen in medical universities of Arak, Hormozgan, and Qazvin; while, medical universities of Lorestan, Semnan, and Shahed faced with negative growth.

**Fig. 3:**
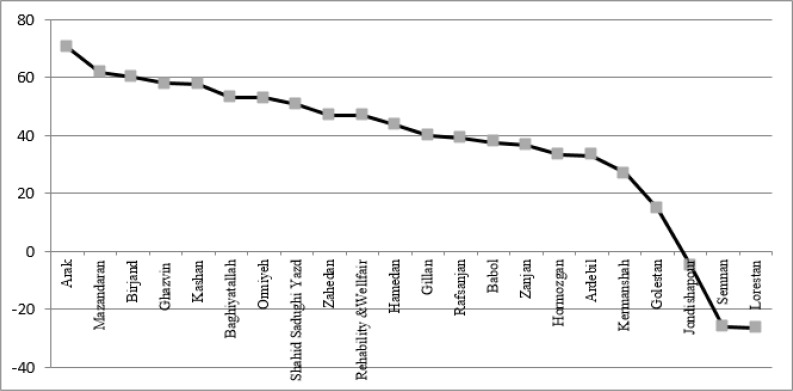
Comparison of growth percentage of webometrics rank of medical sciences university’s Type 2 in Iran during Jan 2012–2015

If we consider the severance of the years, the following universities indicated considerable growth: Rafsanjan with 16% growth in Jan 2015, Ardebil with 55% growth in Jul 2014, Zahedan with 35% growth in Jan 2014, Babol with 16% growth in Jul 2013, Qazvin with 52% growth in Jan 2013, Zahedan with 50% growth in Jul 2012 (Fig. 3).

**Fig. 4:**
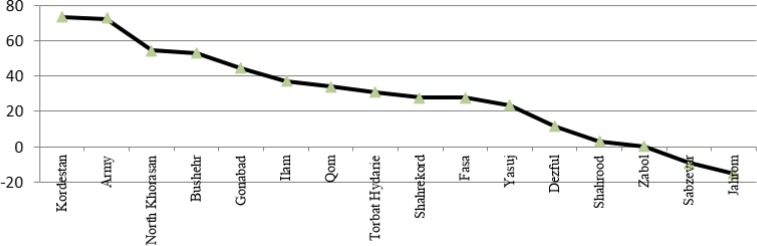
Comparison growth percentage of webometrics rank of medical sciences university’s Type 3 in Iran during Jan 2012–2015

The study of growth rate in type 3 medical universities indicated that the highest growth rate is seen in medical universities of Kurdistan, Army, and North Khorasan; whereas, medical universities of Jahrom and Sabzevar faced with negative growth. If the severance of the years is considered, the following universities indicated considerable growth: Kordestan with 32% growth in Jan 2015, Gonabad with 47% growth in Jul 2014, Fasa with 46% growth in Jan 2014, Qom with 50% growth in Jul 2013, Shahrekord with 51% growth in Jan 2013, Kordestan with 33% growth in Jul 2012. Universities of North Khorasan, Torbat Heydarieh and Dezful have been inserted in webometrics rating system from Jul 2013 (Fig. 4).

## Discussion

Active presence on the Web and publication of research findings are considered as performance indicator of universities and research institutes in the assessment of development. Websites must continually be evaluated in terms of the importance for websites, applicability, and success in achieving the goals. Webometrics assessment of websites of universities and research institutes also has the advantage that puts the spotlight on the current situation and the performance of universities in relation to the web environment from one side. On the other hand, leaders of these organizations try to provide the necessary conditions for their institutions for the active presence on the web according to international standards in order to gain further credibility. Therefore, the universities should care about periodic evaluations. This study elaborates the ranking of the national medical universities. Medical universities of Tehran, Shahid Beheshti, and Isfahan received top ratings in Jan 2015 and medical universities of Kordestan, Torbat Heydarieh, Rafsanjan and Birjand indicated a growth more than 15% compared to the previous rating. Recent results suggest that among 50 universities of medical sciences in the global webometrics ranking, 44% associated with improved global ranking in recent rating. They are generally ranked 37.2% lower than the previous rating. The medical universities of IAU, Shiraz, North Khorasan, Lorestan, Isfahan, Tavanbakshi, Dezful, Zahedan, Hormozgan, Sabzevar, Babol, Kerman, Ilam, Yasouj, Qom, Fasa, Tabriz, Jahrom, Arak, Shahed, Golestan, Zabol, Qazvin, Gonabad, Shahroud, Kermanshah, Ardebil and Hamedan also indicated a decrease.

There are different rating fluctuations in type 1, type 2 and type 3 medical universities considering the above findings from Jul 2012 to Jan 2015. The most promotion at this time was seen in medical universities of Kerman, Qazvin, Fars, Qom, Ardebil and Kurdistan (54%, 52%, 46%, 50%, 55% and 33%, respectively). The most growth in rating of all medical universities is seen in Jan 2014 and the lowest growth is seen in Jan 2015. Changing the method of calculation is one of the reasons to justify this change and negative fluctuation, to calculate the index, previously known as scholar; the results of the evaluation from Google scholar search engine were also used in addition to Scimago.

The ranking fluctuations and downgrading during different time is reported (10). In a recent ranking of webometrics, out of 50 universities, 28 universities have shown a backward.

The findings of the evaluation system of universities and research institutes websites at Regional Information Center for Science and Technology suggest that medical universities of Tehran, Isfahan, and Shiraz are in the top rankings, respectively. Shahid Beheshti University of Medical Sciences is in the fifth ranking conveying the successful results (11).

Although there is a positive correlation between webometrics rating of webometrics system and other university ranking systems, the results of the ranking become somewhat different by using different criteria in the ranking systems (12). HEEACT rating system focuses on current research and ARWU emphasizes the performance of universities in research, outstanding success and timer quantity and quality of university performance (13).

The webometrics rating of universities in Jan 2015 includes significant points, websites of medical universities of Tehran, Isfahan and Shahid Beheshti in the presence index; websites of medical universities of Tehran, Isfahan and Shahid Beheshti in the impact factor index; in terms of access to rich files the medical universities of Tehran, Birjand and Shiraz; and finally the medical universities of Tehran, Tarbiat Modares, and Shahid Beheshti showed the best science ranking index. The Medical University of Tehran exceeds the points both in web presence and number of links involved followed by two universities of Guilan and Mashhad (14). A web studies on medical universities in 2009 showed that Tehran Medical University has the highest number of web pages (15).

Having studied the Universities of Medical Sciences, according to Yahoo and Bing websites the visibility ranking was 13^th^, while the Rich files ranking appeared to be the third nationally (16). The impact level of 15 university websites were studied and indicated that Tehran Medical University owns the biggest websites capacity, whereas Shahid Beheshti has the highest impact level. According to this research, linguistically, the Middle East websites exhibited in Farsi, Kurdish, Turkish, Arabic and Hebrew receive fewer links and users (17).

Even type one medical universities have not received links as well as users via outside their websites. This is indicative of low level of medical universities impact on web, despite their numerous web pages. Other studies confirmed this statement too (6, 16). Impact indicator in the medical university website is the most effective factor in webometrics ranking; this might be due to allocate more site management personnel, presenting useful information and linguistic reasons too. Although, promoting academic content and implementing optimization standards need further attention.

Finally, in order to increase the visibility of university website, it is recommended to provide the citing potentiality through the search engines. Further, accessibility and abundance of Rich Text files will assist the website visibility. In addition, to increase the traffic ranking and visibility, Persian texts need to be translated into English and one person from each educational department or group to be assigned as an advisor or supervisor in order to update the page and/or promote the quality as well as the quantity of the relevant homepage.

## Conclusion

Researchers may not be able to prevent the bibliometrics ranking or efficacy impact trends and policies. The production volume and quality of research conducted by individual researchers, research centers and universities are two of the important criteria contributing wisely to success and economic efficiency. Despite the criticisms and weaknesses brought up for Bibliometrics as well as webometrics criteria, these criteria are considered as a critical performance for this equation. They must be checked carefully and meticulously for authenticity and goal achievement indicators. Therefore, localizing these criteria, development and precise evaluation according to these indices provide new opportunities for national development, especially through online media providers.

## Ethical considerations

Ethical issues (Including plagiarism, informed consent, misconduct, data fabrication and/or falsification, double publication and/or submission, redundancy, etc.) have been completely observed by the authors.
